# Soy and the gut microbiome: a bi-directional relationship shaping nutrition and health

**DOI:** 10.1007/s00394-026-03900-4

**Published:** 2026-02-12

**Authors:** Laura Nolan, Paul W. O’Toole

**Affiliations:** School of Microbiology and APC Microbiome Ireland, University College Cork, Room 447 Food Science Building, Western Road Cork, Cork, T12 K8AF Ireland

**Keywords:** Microbiome, Microbiota, Plant proteins, Soy, Isoflavones, Host-microbe interactions, Dietary interventions

## Abstract

The gut microbiome is a transducer of the health effects of many food ingredients because of its ability to convert them into health-impacting compounds. Plant-forward diets and plant ingredients are recognized as health promoting, with recent added impetus provided by the drive to provide consumers with more sustainable protein sources than meat. Soy-based foods are good protein sources, and they contain a variety of other ingredients such as fibre that can impact the microbiome. This review explores the health benefits reported for soy-containing foods and the role of microbiome involvement in health effects. It describes the bi-directional relationship between dietary soy and the human gut microbiome whereby microbial metabolism transforms soy-derived compounds into bioactive forms that influence host physiology, while soy consumption shapes gut microbiota composition and activity. Factors modulating the health effects of soy such as fermentation, bioavailability, and consumers’ individual microbiome configurations are discussed. Current research limitations, including inconsistent methodologies and a lack of longitudinal dietary intervention studies are addressed, as well as the need for multiple omics approaches in microbiome research. Finally, we present an interdisciplinary perspective of the complexity of soy-microbiome interactions, potential for health promotion, and relationship to sustainability.

## Introduction to microbiome-diet interactions

Human nutrition studies acquired a new dimension with the recognition that the human gut microbiome plays a role in transducing food ingredients into bioactive compounds and metabolites [1, 2]. The gut microbiota is the dynamic ecosystem of microorganisms including bacteria, fungi, viruses that inhabit the gastrointestinal tract, and their genes (microbiome), that play an important role in maintaining physiological functions through interactions with the alimentary, immune, endocrine, and nervous systems, as well as the gut-liver, gut-kidney and gut-brain axes [3]. Alterations in the microbiome are linked with a broad spectrum of diseases, particularly cardiometabolic diseases [4] and type 2 diabetes [5], diseases of immune dysfunction such as Inflammatory Bowel Disease [6], colorectal cancer [7], and diseases of gut-brain interaction (DGBIs) such as Irritable Bowel Syndrome (IBS; [8]). Potential impacts on neurological diseases are also being investigated [9, 10]. For some of these diseases, putative mechanisms have been identified, such as the contribution of microbially-produced branched chain amino acids to type 2 diabetes [11], and the mutagenic effect of genotoxic *Escherichia coli* strains in colorectal cancer [12]. Where a mechanism has not been established but the disease-microbiome association is reproducible, as for many non-communicable multi-factorial lifestyle diseases, a pragmatic approach is to treat the microbiota as an environmental modifier of disease risk [13]. Emerging evidence suggests that prebiotics, gut microbes, and their products may mitigate the effects of mycotoxins in food [14], emphasizing the complexity of these interactions.

Factors that influence microbiota composition and function include age [15], geography [16], antibiotic treatment [17], and habitual diet [18]. Diet-microbiome links are particularly interesting because they allow investigation of the microbiome contribution to a known health effect of a food, which could be beneficial or deleterious. A controversial example of the latter is the conversion of carnitine, choline, or glycine betaine by certain gut microbes, whose genomes encode the necessary enzymatic machinery, to trimethylamine (TMA). This is oxidized in the liver to trimethylamine oxide (TMAO), which promotes atherosclerosis [19], but there are contradictory findings [20]. The mammalian gut is unable to digest complex fibre, a function that is provided by the gut microbiome [21] but which varies significantly between individuals depending on the composition and functional capacity of their gut microbiota [22]. Dietary fibre, which cannto be digested by human enzymes, is converted by gut microbes into short chain fatty acids including butyrate and propionate that have a broad range of health benefits [23]. The activity of the gut microbiota may underpin the well-recognized inverse correlation between fibre intake and colon cancer risk [24, 25].

The proliferation of deeply-phenotyped cohort studies has consolidated the associations between habitual diet, the microbiome and health [26–28], and Plant-forward dietary patterns are most consistently associated with particular microbiota profiles and health effects in these studies. Soy intake is not typically individually identified in the dietary instruments used, but focussed analysis of dietary soy intake in two American cohorts supports its playing a role in microbiota-mediated health benefits (reduced systolic blood pressure [29]). As with many other dietary components, soy protein and the microbiome are expected to exhibit a bi-directional relationship, whereby soy induces microbiota composition shifts, and the resulting microbiota produces soy-derived metabolites, many of which appear to confer health benefits. This review comprehensively explores the complex interplay between soy consumption and the gut microbiome. Our focus will be on recent research findings and high-quality cohort studies. Current methods, perspectives, interdisciplinary research, and limitations will be discussed.

## Properties and comparative analysis of soy ingredients

Soybeans consist of approximately 40% protein (by dry weight), 20% fat, and 30–35% carbohydrates [29, 30]. Fat components are predominantly polyunsaturated, and soy is rich in essential fatty acids [31]. The carbohydrate content includes various mono- and oligosaccharides that represent up to 10% of total content [32] consisting variably of about 5% sucrose, 4% stachyose, and 1% raffinose, all of which are readily metabolized by the gut microbiome. Fibre accounts for approximately 9–16% depending on the variety [33]. Soy soluble polysaccharides have a pectin-like structure with a core dominated by equal amounts of L-rhamnose and D-galacturonate, and comprising a diglycosyl repeat of 15, 28 or 100 units per chain [34]. Chemical analysis of the sugar content of insoluble fibre ranks glucose (3.9% of total carbohydrate), uronic acid (2.5%) galactose, xylose (1.4%) and arabinose (1.4%) as the most abundant monomeric components, with significant differences depending on bean strain e.g. yellow versus green [33, 35]. The high glucose content indicates that cellulose is a predominant polysaccharide in soybeans [33]. Interestingly, one of the key differences between yellow and green soybeans was the higher galactose content in yellow soybeans [33]. This would be expected to have a significantly different impact on the gut microbiota. The effect on polysaccharide polymers of fermentation for soy-containing foods is expected to change their availability to fibrolytic microbes (see below).

A key driver for increasing the plant component of the diets of humans and production animals is the reduction in the carbon footprint attached to animal protein production [36]. However, the individual molecular structures and compositions of plant proteins mean that they may have lower bioavailability and nutritional quality than their composition alone suggests [37]. Soy protein is a high-quality plant-based protein comparable to animal-based proteins [38]. It is particularly high in lysine content, and it has a good balance of branched-chain amino acids [39]. However, like other legumes, soy protein is relatively low in methionine and cysteine content [40]. A common method to rate protein quality is the Protein Digestibility-Corrected Amino Acid Score (PDCAAS; [41]), based on the completeness of the amino acid profile and its digestibility. Plant proteins generally have diverse amino acid profiles but often lack one or more essential amino acids [39]. The soy PDCAAS score is generally considered to be 100% [38] although this value can vary slightly depending on the specific soy product and processing methods used. The protein content of soy by weight depends on its processing state [39], but the data generally support using this legume as a source of dietary [38]protein. Independent of other nutritional or bioactive properties (reviewed below), the fibre and protein content of soy, plus its potential to positively modulate the gut microbiome, render it very attractive for increasing protein intake in older people [42].

Soybeans have long been recognized as being rich in phytochemicals including isoflavones [43], molecules with non-steroidal and phenolic molecular structure. These isoflavones are called phytoestrogens because they can bind to the human oestrogen receptor [44]. The main isoflavones in soy are genistin, daidzin, and glycitin [45] which are are converted in the intestine to aglycones, genistein, daidzein, and glycitein. Daidzein is converted by gut microbes to equol, and so it is possible to classify individuals as “equol producers” or “non-producers” [46]. Fermentation increases the conversion of phytoestrogens to the aglycone form [47], and equol binds to the human oestrogen receptor with greater affinity than daidzein [48] and other aglycones. Thus, microbial activity during both food fermentation and gastrointestinal digestion is a major modulator of the levels of this bioactive in soy. The role of the gut microbiota in altering the bioavailability or bioactivity of food ingredients or their downstream metabolites is formalized in the “metabotype” concept [49]. This emphasizes the importance of the “baseline” microbiome in determining, for example, if a given subject is a responder or non-responder in a food intervention trial. The main metabotype relevance for soy ingredients is the daidzein to equol conversion mentioned above [46], which also describes the stratification of gut microbiomes into those capable of production or non-production of urolithins from polyphenols. Thus, individual variability due to the existence of different metabotypes is likely hampering the identification of significant outcomes in small scale trials.

Glyceollins are produced by soybeans from daidzein in response to stress or fungal attack (reviewed in ref. [50]). Glyceollins are a family of phytoalexins, a compound type that includes resveratrol which is very well studied, whereas soy glyceollins are less well understood on several levels. They attracted attention because of their reported anti-cancer activity (see below), but they also appear to modulate the microbiota. Soybeans also contain saponins which are triterpenoid aglycone structures that may comprise from 0.6% to 6.5% dry weight depending on bean variety and growth conditions [51].

## Health benefits associated with soy consumption and possible microbiome involvement

**Fig. 1:**
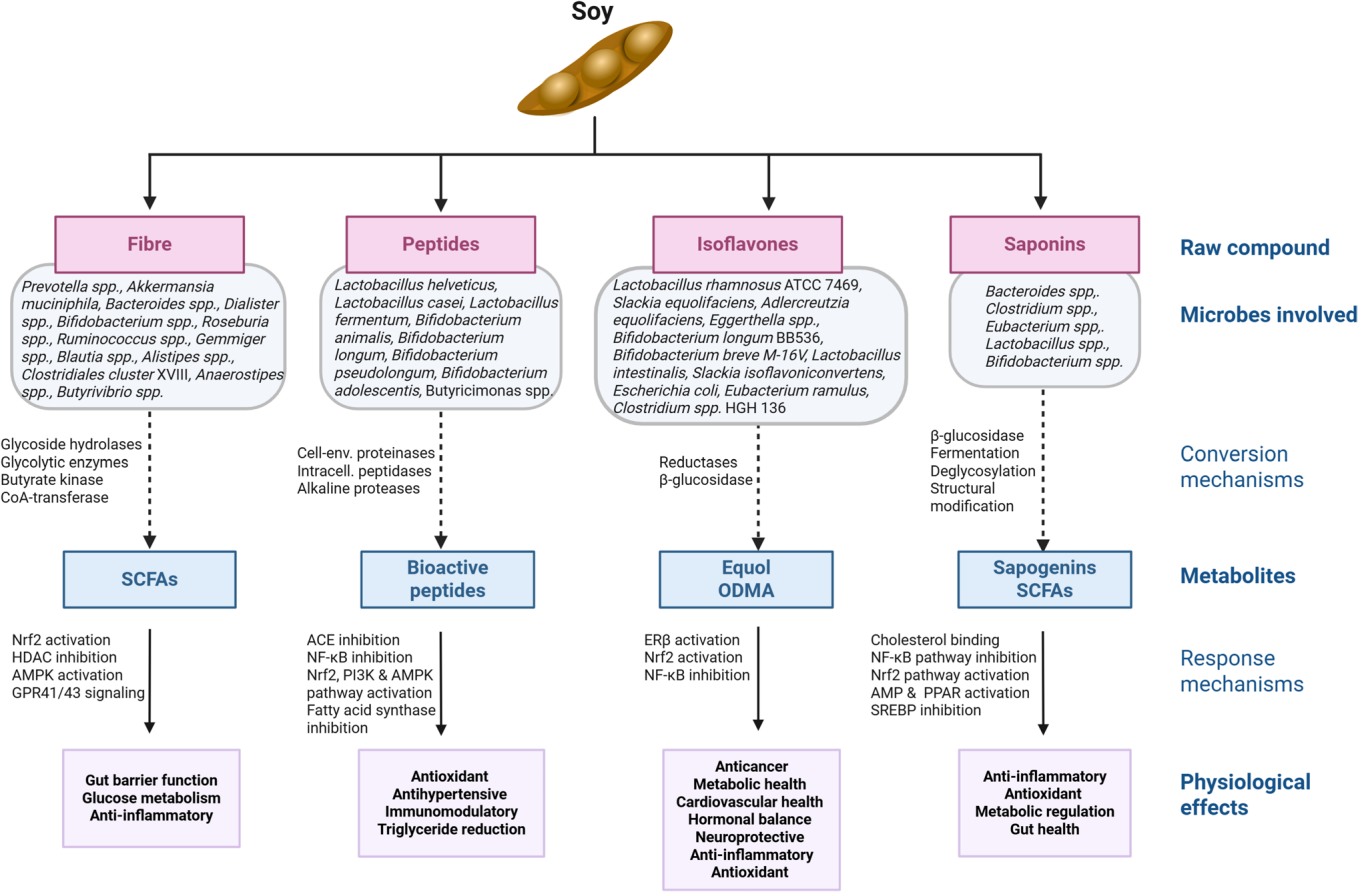
Schematic overview of soy-modulated gut microbiota effects on health. The figure illustrates the raw bioactive compounds found in soy, their microbial-generated metabolites, the enzymes and microbes involved, the ensuing physiological effects, and the mechanisms by which these occur. Created in BioRender. O’Toole, P. (2025) https://BioRender.com/uephrbk

A detailed account of the full spectrum of health benefits associated with soy consumption is outside the scope of this review, and the reader is referred to several studies of this topic [52–56]. Table 1 lists the best described examples among the wide variety of these reported health benefits, if there is literature linking the gut microbiome to that condition, and if there is possible mechanistic overlap or synergy. Figure 1 shows these health effects as the end result of microbial action upon soy, the metabolites produced, and the mechanisms involved in the host response.

**Table 1:** Selected examples of health effects of soy consumption and possible microbiome overlap or involvement

Health condition/system	Bioactivity in soy	Microbiota involvement
Ischemic arterial syndromes	Reduced platelet aggregation and factor VIII levels; increased anti-thrombin levels [54]	Possible synergy through microbiota modulation of vascular phenotype and thrombus formation [57]
Osteoporosis	Vitamin K in fermented soy products modulates osteo-blast/clast differentiation [58]	Possible synergy through effects on hormone production, nutrient absorption, bone marrow generation [59]
Tye 2 Diabetes Mellitus	Unclear; may be due to enhanced glycaemic control via several mechanisms [60]	Possible overlap: Effects on glycaemic control, insulin sensitivity and systemic inflammation are mooted but still unclear [5]
Metabolic syndrome	Unclear; Improvement in body weight, BMI, serum triglyceride profiles with whole soy foods [61]	Possible overlap with microbial mechanisms e.g. SCFA, GPCR-41, -43, GLP-1 and peptide YY effects on metabolism [62]
Cancer (diverse)	Inhibition of cancer cell proteases by soy protease inhibitors [63]; glyceollins, genistein and daidzein act as estrogen receptor modulators and modulate NF-κB signaling [64, 65]; mostly in vitro or animal model data [55]	No obvious overlap; dietary fibre may reduce colon cancer risk by excluding pathobionts via competitive exclusion [66]. Effect of glyceollins on microbiota is reported but provisional
Innate immune response	Mainly anti-inflammatory activity of LAB-fermented products, microbial products could be the effectors [67]	Possible overlap. Well-established property of individual probiotics and gut commensals, their surface macromolecules and metabolites [68]
Elevated blood pressure	Peptide inhibitors of angiotensin-I converting enzyme, found in soy sauce [69]	Mechanistic overlap; some microbes produce ACE I inhibiting peptides when fermenting milk [70]
Cognitive function	Unclear; one study suggests antioxidant and anti-inflammatory activity of aglycone isoflavones [71]	Possible overlap. Fermented soy could produce metabolites that enhance cognitive function, that are produced by the microbiota from other dietary ingredients [72]

Positive effects of soy consumption on metabolic syndrome have been noted by some studies [73, 74]. A hospital-based cohort study of 58,701 participants in Korea investigated the association between intake of Jang (fermented soybean paste) and metabolic syndrome (MetS) risk [75]. Higher Jang intake (≥ 1.9 g/day) was inversely associated with MetS components including waist circumference, body fat, serum glucose concentrations, and hypo-HDL-cholesterolemia [75]. Soy promotes satiety and benefits weight management due to its high fibre content and the release of hormones like cholecystokinin, glucagon-like peptide-1, and peptide YY [62]. The Adventist Health Study-2 followed 96,000 Seventh-day Adventists in the United States and Canada, examining the effects of various dietary patterns on health outcomes. The study found that individuals who consumed soy products regularly had a lower body mass index and were less likely to be overweight or obese compared to those who did not consume soy products [56, 76]. Significantly improved fasting glucose was reported in a meta-analysis of randomized controlled trials [77]. A cross-over trial demonstrated that a soy-based dietary supplement improved blood glucose and insulin levels in type 2 diabetic subjects [78]. Additionally, a review of cohort studies indicated that higher soy intake was associated with a reduced risk of developing diabetes, particularly in women [60]. None of these studies included a microbiome analysis component, unfortunately, and the mechanism(s) involved in health benefits are still preliminary. It is conceivable that soy consumption acts, at least in part, by promoting the growth of gut species that are inversely associated with metabolic disease risk [79]. However, the relatively poor understanding of how the gut microbiota modulates diabetes risk presents a challenge for testing this hypothesis.

Soy protein consumption has been reported to reduce the risk of various cancers especially breast cancer [80, 81]. A 2017 meta-analysis investigated the relationship between soy product consumption (measured by Food Frequency Questionnaire) and the risk of gastrointestinal (GI) cancer [82]. The analysis included 22 prospective studies from 1990–2016, comprising 21 cohort studies and one nested case–control study. A pooled odds ratio (OR) of 0.857 inversely associated soy consumption with overall GI cancer incidence and with gastric cancer [82]. It is not clear what other risk factors were adjusted for in their models. The relationship between soy consumption and colorectal cancer was not significant. Interestingly, the interaction between soy consumption and GI cancer incidence was significant only for women. One could speculate that gender-specific microbiome factors might contribute to this difference [83]. As noted above, soy contains high level of isoflavones [84] that are regarded as phytoestrogens due to their structural similarity to 17-β-estradiol which binds to the oestrogen receptor [85] and can thus affect cancer risk. Soy glyceollins, produced from daidzein, also have anti-cancer activity, primarily against hormone-response tumours such as breast and prostate cancer, consistent with an oestrogen-receptor mediated activity [64]. An interesting recent paper suggested that the inhibition of prostate cancer cell growth in vitro by soy-derived glyceollins was due to effects on the androgen-mediated pathway, but inhibition of tumour xenografts in mice was independent of the androgen-mediated pathway, and instead involved inhibition of tumour cell proliferative marker PCNA [86]. It was suggested that the in vivo effect involved modulation of the gut microbiome, but this analysis was performed using group-specific qPCR primers, and only bifidobacteria were affected (significantly reduced) by glyceollin in the diet. It also incorrectly attributes production of the short-chain fatty acid butyrate to Bifidobacteria [86]. Further investigation is warranted.

There are other mechanisms by which soy consumption could affect cancer development [87]. Peptides derived from soybean meal, such as lunasin and soymorphin, can significantly inhibit the growth of colon, liver, and lung cancer cells in vitro [88] independently of oestrogen receptor binding. Lunasin has been shown to inhibit non-small cell lung cancer cell proliferation in vitro by suppressing phosphorylation of the retinoblastoma protein [89]. Protease inhibitors in soy, such as the Bowman-Birk Inhibitor (BBI), regulate digestive enzymes and display anticancer properties, possibly by preventing extracellular matrix degradation and therefore tumour invasion and metastasis [63]. A recent Phase IIa clinical trial in oral leukoplasia patients showed significantly reduced lesion size in patients treated with BBI [87]. More clinical trials are required, ideally Phase III, to move beyond the current preponderance of pre-clinical data for the anti-cancer properties of soy ingredients.

While there is no obvious microbiome link to the anti-cancer properties of soy, microbial fermentation of soy enhances its nutritional profile and bioavailability, and in some fermentations enhances the release of peptides including lunasin [90]. Microbial fermentation with lactic acid bacteria and yeast (*Saccharomyces boulardii*) increased the bioavailability of soy phytoestrogens [91] (which is not always desirable in food consumed as a staple), and has been reported to significantly reduce the levels of anti-nutritional compounds including protease inhibitors and phytates [92]. The vast diversity of fermentation conditions and microorganisms employed globally, ranging from adventitious cultures on artisanal/traditional foods through to commercial yeast and lactic acid bacteria, contributes to the diversity of bioactive composition and health effects reported for soy-containing products.

Diseases of Gut-Brain Interaction (DGBI) also encompass putative links between the gut microbiome and cognitive function [93]. There are reports from studies that did not investigate the microbiome that consumption of soy-containing foods may reduce dementia risk, improve short and long-term memory, and improve memory retention and verbal fluency [94–96]. These neuroprotective effects have been attributed to soy-mediated increases in neurotransmitter levels, support of synaptic plasticity, reduction of oxidative stress and inflammation in the brain, and enhancement of brain-derived neurotrophic factor levels [97, 98]. A recent study explored how a soy-based diet affects gut permeability and microbiome composition in mouse models of fragile X syndrome and found that a soy protein isolate-based diet *increased* gut permeability [99]. It also altered the caecal microbiome, with changes in the relative abundance of taxa including *Akkermansia muciniphila*, family *Ruminococcaceae* and *Anaerovorax* species. *A. muciniphila* abundance in humans is linked to fibre intake [100] and is generally associated with health benefits [101]. Increased gut permeability is normally considered undesirable, but microbiota alterations could have complex effects in this artificial fragile X model that do not reflect what occurs in humans. The effects of soy-derived microbial metabolites on the gut-brain axis might be a useful model system for exploring nutrition-microbiome interaction.

## Microbiome-soy interaction

The composition and function of the gut microbiota are strongly influenced by habitual diet rather than single dietary components, and in fact the *overall* diet composition correlated more closely with the microbiota composition than individual dietary components, in a study of 1800 adults in the American Gut Project [102]. This is reflected in our experience whereby a 6-month intervention with 20 g prebiotic consumed daily by frail elderly subjects had limited effects on microbiota composition and none on alpha diversity [103], whereas a one-year Mediterranean diet intervention in pre-frail elderly subjects across Europe retained microbiota diversity, promoted specific diet-responsive taxa, and improved or retained multiple readouts of healthy aging [72]. Considering the effect of individuals’ metabotypes on conversion of soy ingredients to bioactive compounds [46] may aid interpretation of studies of the soy-modulated microbiome or the health impact of the soy-modulated microbiome, which would be facilitated by measuring the established dietary patterns of the study cohort.

A preponderance of the studies that investigate the effect of soy on the gut microbiome were conducted in pre-clinical models, and these were recently reviewed comprehensively [104]. These pre-clinical studies are useful exploratory tools and hypothesis generators, but are limited because they were performed in mice or rats harbouring their native microbiota, so-called “conventional” animals. A better alternative is to study germ-free animals that were humanized by faecal transfer from subjects of a particular phenotype, as we have performed for studying the prebiotic response of the gut microbiota from frail elderly subjects [103]. The rodent microbiota is very different to that of humans, and differs between commercial animal providers [105], plus there is a large translational gap from rodent-based studies to human validation [106]. Some of the evidence linking soy consumption to the human gut microbiome is therefore indirect evidence, gleaned from overall diet patterns. For example, the effect size of soy product intake on the gut microbiome is in the top 25 rank-order features in the Flemish Gut Flora Project, and soy product intake has the same effect size on the micorbiome as that of total fruit consumption [107]. Another approach to test the effect of soy ingredients on the human gut microbiome are in vitro culture models. Adding isoflavones in a model seeded with faecal microbiota of equol-producing menopausal women increased the relative abundance of *Collinsella*, *Faecalibacterium* and members of the Clostridium clusters IV and XIVa [108]. However, human feeding trials, ideally with well-defined soy-containing foods are required to determine the effect of soy consumption on the human gut microbiome. Relatively few such trials have been performed. Table 2 lists examples in reverse chronological order.

**Table 2:** Human dietary interventions that tested the effect of soy on the human gut microbiota

Study product	References	Subject number/description	Microbiota alterations	Health effects	Comment
Cheonggukjang (fermented soy product)	[109]	60 postmenopausal Korean women	Reduced proportion of phylum Firmicutes	Menopausal symptom relief and reduced blood glucose only in “high beneficial bacteria” product group	Study product was stratified based on fermentation process and product “beneficial bacteria” content. Microbiota effects were related to this, and only presented at phylum level
Black soymilk (instead of pork in diet)	[110]	8 test, 8 control pre-diabetic/obese Taiwanese adults	No changes in alpha- or beta-diversity; increased relative abundance of *Butyricicoccus* and SCFA producers; lower abundance of *Rothia* spp.	Reduced oxidative stress and lower levels of glycative stress markers	V3-V4 16S amplicon sequencing limits resolution
Fermented versus unfermented soy milk	[111]	56 test, 56 control healthy Japanese adults with low Bifidobacterium count	Increased abundance of lactobacilli and bifidobacteria, *Faecalibacterium* in both groups, more pronounced in fermented soy group; increased *Clostridiaceae* and *Enterobacteriaceae* in non-fermented group	Higher levels of faecal SCFAs in fermented soy group; improvement in defaecation habits in both groups, achieved earlier in fermented soy group	V1-V2 16S amplicon sequencing was supplemented by targeted qPCR
Commercial fermented soybean beverage	[112]	Lean (10) and obese (9) participants in USA	Increased abundance of *Bifidobacteria* and *Blautia* in lean subjects; increased oral abundance of *Veillonellaceae* in lean subjects; study product significantly increased Fusobacteria in obese participants	Not tested	V4 16S amplicon sequencing
Fermented soy milk (FSM) plus *L. casei* plus aglycones, versus unfermented soy milk	[113]	30 test, 30 controls in Japan	*Lactobacillaceae* significantly increased, *Bifidobacteriaceae* trended upwards, *Enterobacteriaceae* and *Porphyromonadaceae* significantly lower in FSM group	Both groups had improved skin condition and significantly increased levels of urinary isoflavones	V1-V2 16S amplicon sequencing
Soy isoflavone concentrate tablet, 80 m/day	[114]	16 menopausal women for 6 months, in Spain	Increased abundance of *Lactonifactor longoviformis, Faecalibacterium prausnitzii, Bifidobacterium sp., Ruminococcus sp*., isoflavone metabolizers; *Clostridium leptum* and *C. coccoides* abundance increased in equol producers	Not tested	PCR-DGGE* and qPCR
Soy bars containing isoflavones	[115]	17 postmenopausal women in USA	Significant increases in Bifidobacterium after soy consumption; Bifidobacterium and Eubacterium were significantly greater in equol vs non-equol producers	Correlations detected between urinary isoflavones and isoflavone metabolites with microbiota	Microbiota was studied by PCR-DGGE* and pyrosequencing of 16S V4 region
Low glycinin soymilk (LGM), conventional soymilk (S) or bovine milk	[116]	64 overweight and obese men in USA	Total bacteria increased in all treatments; *Bacteroides*-*Prevotella* increased in LGM; *Firmicutes*: *Bacteroidetes* ratio decreased in LGM and S groups; *Eubacterium* and *Clostridium* were more abundant in the S group	Not tested	qPCR for total bacterial load and quantitation of Bacteroides-Prevotella, Bifidobacterium, and Lactobacillus; Short-read sequencing of V1-V3 region. Microbiota of all 3 groups separated by beta-diversity analsysi
Non-fermented soybean milk (NFSM) and fermented soybean milk (FSM)	[117]	10 healthy subjects, 21–25 years old in Japan	Bifidobacteria and lactobacilli were more abundant in both groups, Clostridia were less abundant	Not tested	Bacteriological culture on selective media
Non-fermented soybean milk (NFSM) and fermented soybean milk (FSM), cross-over	[118]	28 healthy adults in China	Bifidobacterium spp. and Lactobacillus spp. Increased; higher ratios of *Bifidobacterium* spp. and *Lactobacillus* spp. to *Clostridium perfringens* (P < 0.05)	Not tested	Bacteriological culture on selective media

*DGGE: Denaturing gradient gel electrophoresis

The most commonly reported microbiota alterations associated with soy consumption are increased relative abundance of presumptively beneficial bacteria such as bifidobacteria and lactobacilli (Table 2), and an altered ratio of the phyla *Firmicutes* to *Bacteroidetes*, a relatively crude measure of phylum-level microbiota abundance (the so-called F/B ratio; these phyla are now renamed as *Bacillota* and *Bacteroidota*, respectively). Bifidobacteria are well characterized human gut commensals, some species of which are linked to improved gut health and modulation of inflammation [119]. Neonatal isolate species are genomically equipped to metabolize human milk oligosaccharides, whereas species from adults are highly saccharolytic and encode enzymes to metabolize plant polysaccharides [120]. Their outgrowth after soy consumption is therefore likely to be in response to the fibre content of the soy. Lactobacilli are well characterized for their role in food production and promoting gut health as probiotics [121] but most gut species are not endowed with genes encoding complex polysaccharide metabolism, and they may be cross-feeding on oligosaccharides released by bifidobacteria or *Bacteroides*-*Prevotella* taxa reported in one study [116]. However, the focus on relative abundance of lactobacilli and bifidobacteria in many studies, and the F/B ratio epiphenomenon, are not particularly helpful in moving the field forward. Most of the studies in Table 2 are statistically underpowered and thus unable to detect effects of study product against the baseline differences in the participants’ microbiota. None of them treated the pre-intervention habitual diet and corresponding microbiome as a variable in statistical models. Ironically, it was one of the smaller studies that stratified 17 participants as equol producers or non-producers based on HPLC analysis of urine samples [114]. Lastly, some of the older studies naturally used culture-independent methodologies with limited phylogenetic resolution, but even recent publications based on 16S V3-V4 amplicon sequencing produce sequence reads that cannot all be assigned to species level, and that cannot detect strain-dependent gene content. For example, different strains that are all identified at 16S level as “*Prevotella copri*” can have gene content that differs at strain level and which have dramatically different interaction with habitual diet or antibiotics [122]. Metagenomic sequencing and rigorous pathway analysis of the shotgun metagenome of trial participants, ideally supplemented by faecal metabolomics, will be required to detect genes and gene products enriched in the soy treatment arm.

## Health impacts of a soy-modulated gut microbiome

*Fibre.* Notwithstanding the paucity of microbiome data from human intervention trials, sufficient evidence has accumulated from in vitro experiments, pre-clinical models, cross-sectional cohort analyses and bacteriological culture to assemble an overview of how soy ingredients alter the microbiome, what metabolites are produced, and what the likely physiological effects are (Fig. 1). With regard to soy fibre utilization, *Prevotella* species, which are more prevalent in individuals consuming high-fibre, plant-based diets, are modulated by soy intake [116]. These bacteria produce short chain fatty acids (SCFAs) such as succinate and propionate and have been associated with improved glucose metabolism and reduced inflammation, although their effects can vary depending on the overall dietary context [122, 123]. The baseline proportions of *Bacteroides* spp. and *Prevotella* spp. are important for determining how the microbiota reacts to fibre interventions [124]. SCFAs including butyrate trigger a broad range of anti-inflammatory responses (Fig. 1) through well described mechanisms (reviewed in ref. [125]).

*Akkermansia muciniphila*, a mucin-degrading bacterium associated with metabolic health [101], may be indirectly promoted by soy through its fibre content and the overall improvement in gut microbiota diversity [126]. This species produces acetate and propionate, and *A. muciniphila* is linked to enhanced gut barrier function, reduced metabolic endotoxemia, and improved insulin sensitivity [127]. *A. muciniphila* has been shown to modulate innate immune activity in model systems [128], to have anti-inflammatory activity, and to modulate glucose metabolism [129]. Not indicated in Fig. 1 are oligosaccharides released from soy fibre. *Lactobacillus* and *Bifidobacterium* genera and species are particularly stimulated by fermented soy products such as miso, tempeh, and natto, and soy oligosaccharide prebiotics [130]. Both genera metabolise dietary tryptophan into indole derivatives like indole-3-aldehyde and indole-3-lactic acid. These compounds activate the aryl hydrocarbon receptor (AhR) in intestinal epithelial cells, potentially leading to enhanced mucosal immunity, improved gut barrier integrity, reduced inflammation, and improved insulin sensitivity [131, 132].

*Peptides*. Soy protein β-conglycinin is a major component of soy protein that showed cardioprotective properties in a pre-clinical model which appeared to be mediated by gut microbiome modulation and enhanced SCFA production [133]. The responsive taxa were genera *Butyricimonas*, *Marvinbryantia*, and *Anaerotruncus* [133]. Proteolysis of soy protein (during fermentation or by digestive enzymes) releases peptides that are further converted to an array of bioactive peptides by diverse gut bacterial taxa (Fig. 1). The proteolytic gut species are relatively poorly described, and published evidence is mostly based on proteolysis that occurs during fermentation, and the microbes responsible for peptide release were not investigated in many of these studies. The most frequently reported properties for peptides derived from soy are angiotensin-converting enzyme (ACE)-inhibition [134], and reduction of serum cholesterol [135], reviewed in ref. [55]. In traditional soy fermentations, a wide array of microbes is often present including strains of *Bacillus* spp., *Streptococcus* spp., *Lactobacillus* spp., *Rhizopus* spp., *Candida* spp. and *Aspergillus* spp. [136–138]. In fermentation models, particular strains of *Lacticaseibacillus casei* and *Limosilactobacillus fermentum* have been shown to release peptides with ACE-inhibitory activity, by MALDI-TOF spectrometry during soy fermentation [139]. Release of bioactive peptides from soy is likely carried out by lactic acid bacteria that have a PrtP/PrtH-like cell envelope proteinase on their cell surface [140]. Soy peptide administration can ameliorate barrier function and reduction of microbiome alpha diversity in a mouse model treated with the cancer chemotherapy agent irinotecan [141]. Reduced toxicity of irinotecan was linked to increased tight-junction protein production induced by the soy peptides [141]. More detail on the soy peptide composition used and independent replication in other models is desirable. Several studies suggest that soy peptides can reduce the levels of cholesterol and triglycerides (reviewed in ref. [142]). The mechanisms reported are inhibition of cholesterol synthesis, inhibition of fatty acid synthase, and promotion of LDL uptake.

As shown in Fig. 1, soy-derived peptides also affect signalling pathways that control innate immunity and inflammation through multiple signalling pathways, particularly the TLR4/MAPK/NF-κB axis, integrin signalling, and the PI3K/Akt/mTOR pathways [143–147]. Much of this data comes from cell lines, and the detailed mechanisms are not known.

*Isoflavones*. Isoflavones constitute the most abundant type of phytochemical in soy, and are converted by gut microbes into two main bioactives, equol and O-desmethylangolensin (ODMA). In one study, the proportions of tested individuals in the US whose gut microbiome produced equol and ODMA were estimated at 41% and 80% respectively, and obese individuals were almost three times more likely to be ODMA producers [148]. Equol is produced by a range of bacterial taxa including strains of *Slackia equolifaciens* [149], *Slackia isoflavoniconvertens* [150], *Asaccharobacter celatus* [151], *Enterorhabdus mucosicola* [152], and *Adlercreutzia equolifaciens* [153], plus some additional well characterized lactobacilli and bifidobacteria shown in Fig. 1. Equol production requires the sequential activity of three reductase enzymes which are not present in all *Adlercreutzia equolifaciens* strains explaining strain variability in equol production. [154]. Despite the prevalence of this activity in the population, ODMA producing bacteria are much less well identified and understood. *Eubacterium ramulus* can convert daidzein to ODMA [155]. *Clostridium* spp. HGH136 sp. was reported to cleave the C-ring of daidzein to produce ODMA [156] but there has been no follow-up characterization.

Equol production status can be an important factor and confounder in studies of the health effects of soy. For example, in an intervention trial comparing the effect of 12-month diet supplementation with soy protein containing 99 mg per day of isoflavones with milk protein (placebo), there was no difference in blood pressure between treatment groups, but systolic and diastolic blood pressure decreased and endothelial function improved in the equol producer subgroup of the soy protein treatment group [157]. Thus, the mechanistic links between isoflavone metabolites and health warrant much greater attention. A recent study from China suggests another microbiome link with the equol-adiposity axis [158]. Based on cross-sectional and longitudinal analysis of 2959 adults over six years, urinary equol levels were inversely correlated with obesity factors BMI, serum triglycerides, and proportion of fat mass. The microbiota of equol producers displayed higher alpha diversity and some differentially abundant taxa. Some acylcarnitine metabolite levels associated with adiposity (palmitoylcarnitine, oleylcarnitine 18:1, and stearylcarnitine) were inversely associated with equol production status. The authors conclude that equol production is protective against obesity, possibly mediated by the gut microbiome and acylcarnitines [158]. The microbiota differences reported between equol producers and non-producers are statistically significant but not very biologically convincing until pooled into a 20-taxon “microbiota score”, and none are equol producers themselves, but they could theoretically still be affecting acylcarnitine levels. Further investigation of this interesting system is warranted.

As for soy-derived peptides, much of the research identifying health effects of isoflavones is based on cell lines, particularly macrophages, dendritic cells, endothelial cells and epithelial cells, and the key signalling pathways affected are NF-κB, MAPK, STAT-1, TLR4, AMPK, and dendritic cell maturation [159–164].

*Saponins*. A broad range of health effects of saponins in humans have been reported, but mechanistic data is of variable quality [165, 166]. They have been described as modulating serum triglyceride levels, glucose response, and cancer risk [167]. The cellular mechanisms that underpin health effects have been explored in a variety of cellular and pre-clinical models. Soyasaponins (A1, A2 and I) were shown to inhibit reactive oxygen species-mediated activation of the PI3K/Akt/NF-kB pathway in a manner linked to the ability of saponin to reduce Reactive Oxygen Species (ROS) production [168]. This saponin moiety has also been shown to inhibit the mitogen-activated protein kinase (MAPK) signalling pathway that regulates inflammatory and oxidative stress response [169]. Dietary soy saponins activate the AMP-activated protein kinase (AMPK) pathway that controls glucose and lipid metabolism [170], and they also inhibit intestinal α-glucosidase [171] which delays glucose absorption, directly impacting postprandial glucose control. Soy saponins improved atherosclerosis in a mouse model, whereby atherosclerotic lesions in ApoE −/− mice fed a high-fat diet were significantly reduced in the aortic root and innominate artery, accompanied by expression changes in TLR4 and MyD88 genes and reduced NF-κB p65 and IκBα phosphorylation [172]. Testing for the replication of these effects in humans is warranted.

Fermentation of soybeans enhances the bioavailability and biological activity of saponins [173]. Diverse microbial glycosidases such as glucosidase, glucuronidase, fucosidase, and xylosidase removal sugar moieties attached to saponins, increasing their bioavailability and activity (reviewed in ref. [167]). Bacterial taxa that carry out this activity are reported as *Lactobacillus* spp., *Bacteroidota* (*Prevotella oris*, *Bacteroides* spp.), *Eubacterium* sp., *Actinobacteria* [now *Actinomycetota* (includes *Bifidobacterium* spp.)], *Streptococcus* LJ-22,, and *Proteobacteria* [167]. Saponins of other plants are well characterised (e.g. ginseng) and are known to be converted into secondary metabolites by the gut microbiome [174]. Data from pre-clinical models suggests that saponins have broad effects on the microbiome. Feeding a saponin-supplemented diet to Chinese turtles reduced the relative abundance of *Lactobacillus*, *Bifidobacterium*, *Bacillus*, *Helicobacter*, and *Bacteroides* species while at the same time induced the expression of key inflammatory response genes [175]. Maternal nutritional supplementation of chickens with soyasaponin promoted transfer of gamma amino butyric acid (GABA)-producing bacteria to their chicks, and was linked to improved intestinal health in the chicks [176]. The gut microbiome of mammals converts the phytochemical soyasaponin into soyasapogenol (sapogenol) [177]. Microbiome-generated sapogenol in mice induces a cytochrome P450 enzyme in the liver that inactivates the anti-cancer drug alpelisib, which targets phosphatidylinositol 3-kinase (PI3K) signaling [178]. Depleting the microbiota by antibiotics, or removing saponins (but not isoflavones or macronutrients) from the mouse chow, restored the anti-cancer effect of alpelisib [178]. This example illustrates the potentially extraordinary complexity of diet-microbiome-host-drug responses involving phytochemicals in the diet.

## Confounders and gaps in soy-microbiome research

Microbiome research in general faces several challenges. A primary one is inconsistent laboratory and analysis methods used by different research groups, rendering comparisons difficult and/or unreliable [179]. However, optimized protocols are already available for DNA extraction [180], and unform sequence analysis pipelines are available for 16S amplicon [181] and shotgun metagenome data [182], that in our hands, have made comparison between global datasets feasible and informative [183]. Cost-reductions in short-read metagenomic sequencing and long-read sequencing will allow sequencing the microbial metagenome in greater depth and allow assembly of metagenome-assembled genome or MAGs that will solve the strain-specificity issue mentioned above, whereby specific metabolic genotypes are lost under a common phylogenetic label.

On the broader experimental design side, small sample sizes, pre-trial diet variability, and lack of long-term follow-up can affect study outcomes. Trial size is usually restricted by available budget and improvement may not be possible. Nevertheless, detailed advice for designing sufficiently powered trials is available, including provision for power calculations based on microbiome-based or host response-based primary objectives [184]. Administering a dietary instrument at trial baseline such as a one-week food diary or validated food frequency questionnaire would generate data that might assist in interpreting responders or non-responders, ideally as a primary factor in the main experimental hypothesis. The reliance on self-reported consumption can be mitigated by a new approach called Metagenomic Estimation of Dietary Intake (MEDI) [185], which quantifies food-derived DNA in human faecal metagenomes to estimate dietary intake. This and related metabarcoding methodology [186] offer a non-invasive, objective alternative to traditional dietary assessment methods, providing a powerful way to measure diet and microbiota composition from the same sample. MEDI can detect food items even when present at low abundances [185, 186]. In the case of interventions involving soy or polyphenols, determination of equol producer status at baseline (e.g. by HPLC analysis of urine) would be beneficial, provided such status is included as a variable in the experimental design. Different compositions and processing steps of soy used in a dietary intervention, whether or not they were fermented and by what organism, and the amounts consumed also impact results and should ideally be standardized to allow comparison between studies.

Trials are often short-term randomized controlled trials (RCTs) and therefore fail to capture the dynamic nature of microbial adaptation and the potentially cumulative effects of soy intake. The controlled environments provided by gnotobiotic animal models and in vitro fermentation systems are useful for hypothesis-driven mechanistic studies, but findings can be challenging to translate to humans. Large-scale longitudinal studies are optimal as they track dietary and microbiome changes over time and aim to capture the complex and diverse interactions between diet and microbial communities across populations. Multi-omics approaches, including meta-transcriptomics, meta-proteomics, and metabolomics, are increasingly being adopted [187, 188]. These methods offer higher taxonomic resolution, functional insights, and a more comprehensive view of host–microbiome interactions. Evolving microbial ecology techniques such as spatial metagenomics, single-cell microbiome analysis, and high-throughput culturomics hold promise, and comprehensive protocols are becoming available [189, 190]. Artificial intelligence and machine learning will enable predictive modelling and personalized interventions [191, 192], and synthetic microbiotas and engineered probiotics may offer targeted therapeutic applications [193]. Longitudinal and personalised microbiota monitoring may become possible, enabled by novel biosensors [194].

Future research on soy and the microbiome could also include safety surveillance of artisanal products, exemplified by a recent study of three major kinds of traditional Chinese fermented soybean [195]. As well as a negative correlation between levels of both *Lactobacillales* and *Bacillales* with potentially pathogenic *Enterobacterales* in soy foods, there was clear evidence that two pathogenic *Klebsiella* strains in the gut of Chinese individuals may originate from fermented foods [195]. Furthermore, the study identified multiple examples of antibiotic resistance genes in the soy food product microbiome, which showed striking levels of residue identity to homologous genes in the gut metagenomes of Chinese individuals. Genes conferring antibiotic resistance in the enteric pathogen *Enterobacter hormaechei* strain present in fermented soy products were found disseminated in multiple gut bacterial species, indicating the likelihood of cross-species horizontal gene transfer [195]. Commercial food products are sold under stringently enforced safety and surveillance criteria in most jurisdictions, and most artisanal products do not cause food-borne diseases, but microbiome analysis can enable less acute risk factors such as antibiotic resistance to be monitored.

## Interdisciplinary perspectives and sustainability

*Precision medicine*. One possible objective of studying the soy-microbiome relationship is to be able to offer soy-based interventions to those most likely to benefit, supported by a robust evidence base. Soy is being actively explored in the field of precision medicine, particularly in the areas of cancer care and biomedical materials. The therapeutic potential of soy isoflavones seems promising in hormone-related cancer management, especially breast cancer. Organisations like the American Institute for Cancer Research are investigating the potential contribution of foods including legumes to treatment of cancers and to integrate it into personalized cancer care strategies [196].

*Public health*. Food science bridges agricultural production, nutritional biochemistry, and human health. As previously discussed, the processing and preparation methods of soy impact the bioavailability of its beneficial compounds and subsequent effects on the microbiota, with fermentation being particularly impactful [197]. Soy-based functional foods and prebiotic formulations are becoming more common, but there is a need to confirm the health benefits of soy protein before public health recommendations can be confidently made. The national health agency of France (ANSES) recently issued a recommendation to limit daily isoflavone intake, and to progressively remove soy-based products from catering outlets such as schools, to limit habitual dietary intake of soy and thus reduce isoflavones intake in potentially vulnerable populations (https://www.anses.fr/fr/content/eviter-les-isoflavones-dans-les-menus-des-restaurations-collectives). This contradicts meta-analyses e.g. ref. [198] and recommendations to consume soybeans by major commissions [199]. Such a situation confuses consumers and stakeholders, so we believe urgent clarification of isoflavone health effects is required.

Other public health policies recommend low-fat milk as a replacement for sugar-sweetened beverages (SSBs) to tackle obesity and diabetes, backed by studies showing that this reduces weight, diabetes risk, liver fat, triglycerides, and blood pressure [200]. However, the benefits of soymilk alternatives remain unclear. The Soy Treatment Evaluation for Metabolic health (STEM) trial aims to provide evidence by comparing the effects of 2% soy milk and 2% cow’s milk as substitutes for SSBs on liver fat and key cardiometabolic indicators in at-risk individuals. This trial is in progress at the time of writing [200]. Several ongoing trials funded by the Soy Nutrition Institute (SNI) Global are investigating the impact of soy on the microbiome in both children and adults [201].

*Biotechnology to enhance soy products.* Biotechnology is employing microbial fermentation and synthetic biology, aiming to transform soy into enhanced functional foods for gut health and personalized nutrition. With rising demand for plant-based milk, fermented soy milk offers both nutritional and sensory benefits. A 2024 study found that soy milk fermented with two specific strains (*Lactiplantibacillus plantarum* MTCC 25432 and 25,433, and *Lactobacillus acidophilus* NCIM 2902) showed strong prebiotic activity, increased levels of bioactive compounds, and high probiotic viability under gut-like conditions, compared to mono-fermented or non-fermented product [202]. The co-fermented product had the highest prebiotic effect and received the best sensory ratings [202]. The functional fermented soy product Q-CAN Plus (referred to in Table 2), is reported to positively modulate both the oral and intestinal microbiota and to potentially improve metabolic and inflammatory profiles [112]. The processing and fermentation details of this commercial product are not publicly available, but it has also been reported as improving cognitive function [71]. Clarification of the bioactive compounds and biological mechanism for this claimed activity is desirable.

Soy-based cheeses have previously faced technological challenges for manufacturers in the replication of the texture and functionality of dairy cheese. Hybrid cheese using soy protein and genetically engineered soybean-derived casein has addressed this issue, and the formulation also offers nutritional and sustainability benefits. It increases plant protein intake, enhances meltability and stretch, and is claimed to support sustainable food production by reducing reliance on animal agriculture [203]. At the other end of the spectrum, genetic modification could be used to enhance the organoleptic properties of soy. Soybeans may contain triterpenoid saponins that impact health benefits and bitterness. The cytochrome P450 enzyme system regulates saponin metabolism, and genetic engineering of this enzyme in the soy plant could modify this pathway to enhance nutritional value and improve taste [204].

*Sustainability*. Soy protein production has a significantly lower environmental impact compared to animal protein, because it generates lower greenhouse gas emissions, requires less land, and consumes less water [205, 206]. Animal-based meat accounts for disputed proportions of food-related greenhouse gas emissions, but around 14% according to the UN FAO [207]. Despite providing only about 20% of global food energy, meat and dairy production use 70% of agricultural land and 40% of arable cropland, while livestock production also accounts for over 25% of global freshwater use (reviewed in ref. [208]). Soy is often grown in large-scale agricultural systems and productivity can be enhanced by understanding and manipulating the soil microbiome [209]. The current EU project Microbiomes4Soy aims to improve the environmental impact of soya bean production, explore the effects of soya bean consumption on the human gut microbiome and contribute to the development of novel sustainable plant-based fish feeds for the aquaculture sector [210]. A recent study explored how gut bacteria in Atlantic salmon metabolize bioprocessed soybean meal, addressing digestion challenges posed by plant-based protein sources [211]. Two novel strains related to *Prevotella paludivivens* and *Acetoanaerobium sticklandii* were enriched in soybean meal cultures, as well as probiotic-associated microbes. These findings could help improve plant-based salmon diets, reducing reliance on fishmeal and promoting sustainable aquaculture while enhancing fish health, growth, and disease resistance [211].

*Culture*. From a cultural standpoint, the understanding of the relationship between soy and the microbiome is deeply intertwined with evolving ideas about food, health, tradition, and identity. Historically, soy has deep cultural roots in East Asian cuisines, where fermented soy products have long been valued for their flavour and health benefits [212]. These traditional practices reflect a cultural understanding of food as medicine, an idea that aligns closely with modern microbiome science. In Western countries, soy has undergone a transformation from a niche health food to a mainstream dietary component, especially within plant-based movements, reflecting changing cultural values around sustainability, wellness, and ethical eating [38]. The gut, and by extension foods like soy that influence it, is not just a biological site but a cultural and political one. It is increasingly seen as a space where modern anxieties about purity, control, and identity play out. For example, the popularity of “clean eating” and probiotic-rich diets often intersects with cultural narratives about self-discipline, gender, and wellness [213]. Furthermore, the cultural framing of soy differs significantly between global regions. In some places, it is celebrated as a symbol of health and longevity while in others it is scrutinized due to concerns about GMOs or hormonal effects [214]. These perceptions influence not only consumption patterns but also how microbiome-related benefits of soy are communicated and understood. An appreciation by scientists of these cultural and societal issues could enable more effective engaged research on the soy-microbiome-health axis.

## Data Availability

No datasets were generated or analysed during the current study.
